# PD-L1 Testing in Urothelial Carcinoma: Analysis of a Series of 1401 Cases Using Both the 22C3 and SP142 Assays

**DOI:** 10.3389/pore.2022.1610260

**Published:** 2022-04-11

**Authors:** Harriet Evans, Brendan O’Sullivan, Frances Hughes, Kathryn Charles, Lee Robertson, Philippe Taniere, Salvador Diaz-Cano

**Affiliations:** Molecular Pathology Diagnostic Service, Queen Elizabeth Hospital Birmingham, Birmingham, United Kingdom

**Keywords:** PD-L1, urothelial bladder carcinoma, predictive marker, Pembrolizumab, Atezolizumab

## Abstract

Immune checkpoint blockade (ICB) drugs are a novel, effective treatment for advanced urothelial carcinoma. Worldwide, several different ICB drugs are approved, each developed and clinically validated with a specific PD-L1 compound diagnostic assay. As a result, PD-L1 testing workflows in routine practice are complex: requiring multiple assays across two platforms, with each assay having a different method of interpretation. Our service tested 1,401 urothelial carcinoma cases for PD-L1 expression, using both the 22C3 PharmDx assay (required prior to Pembrolizumab therapy) and SP142 assay (required prior to Atezolizumab therapy). Of the 1,401 cases tested, 621 cases (44%) were tested with both the 22C3 PharmDx and SP142 assays, 492 cases (35%) with 22C3 PharmDx only, and 288 cases (21%) with SP142 only. Each assay was used and interpreted according to the manufacturer’s guidelines. The rate of positivity we observed was 26% with the 22C3 assay and 31% with the SP142 assay, similar to the pre-licensing studies for both drugs. The discrepancy observed between the assays was 11%, which reinforces the requirement for utilisation of the correct assay for each agent, and limits potential cross-utility of assays. This aspect must be considered when setting up a PD-L1 testing strategy in laboratories where both Pembrolizumab and Atezolizumab are available for the treatment of urothelial carcinoma but also has broader implications for testing of other cancers where multiple ICB drugs and their respective assays are approved.

## Introduction

Immune checkpoint blockade (ICB) drugs have emerged as an effective treatment for many cancers and their use is approved across cancer types. Each ICB drug has been developed alongside a specific PD-L1 companion diagnostic assay. These PD-L1 assays have been designed and validated by clinical trials for specific use in different disease-drug combinations. They use different PD-L1 primary antibody clones, immunohistochemistry (IHC) platforms and protocols, and have different scoring algorithms [1,2].

As a result, when testing laboratories decide which PD-L1 tests to implement in routine practice; they must consider data on both clinical and technical validation. There is little evidence in the literature on the clinical equivalence between the various compound diagnostics. Therefore, it would be hard to justify using a compound diagnostic test other than the one shown to be clinically validated in trials (whether an alternative compound diagnostic test or a laboratory-developed PD-L1 IHC protocol). Furthermore, ICB drugs can be both toxic to patients and expensive for healthcare providers which necessitates accuracy in selecting patients suitable for ICB therapy.

In order to deliver an exhaustive PD-L1 testing service in solid tumours, laboratories have to implement PD-L1 assays on both the Dakolink48 and the Ultra platforms and validate the four companion diagnostics assays, each on the relevant platform (22C3 PharmDx and 28.8 PharmDx on Dakolink 48; SP142 and SP263 assays on Ultra platform). Furthermore, as the readout and the scoring algorithm varies both between and within tumour types, there must be appropriate training for reporting pathologists and scientists.

Worldwide, five ICB drugs are currently approved by the Food and Drug Authority (Atezolizumab, Avelumab, Durvalumab, Nivolumab and Pembrolizumab), and three by the European Medicines Agency (EMA) Atezolizumab, Nivolumab and Pembrolizumab) for the treatment of advanced urothelial cancer following chemotherapy. Furthermore both Atezolizumab and Pembrolizumab are EMA approved for the first line treatment of in advanced urothelial cancer where patients are ineligible for cisplatin containing chemotherapy [3,4]. Currently, only Atezolizumab is NICE approved in urothelial cancer, for use in both cisplatin-ineligible patients and for treatment following platinum-containing chemotherapy [5,6]. When being used as a first line drug, both NICE and the EMA specify that the use of Atezolizumab or Pembrolizumab is based on specific PD-L1 cut off values being met and so PD-L1 assessment is mandatory before prescription in these scenarios [5,7,8].

Based on clinical trial data, when using the 22C3 PharmDx assay (the assay approved for PD-L1 testing prior to Pembrolizumab prescription), a positive PD-L1 test result is defined as a combined positive score (CPS) including tumour and immune cells above 10 (CPS >10) [9,10]. However, when using the Ventana SP142 assay (the approved assay for use prior to Atezolizumab prescription), a positive PD-L1 result is defined as PD-L1 positive immune cells (IC) staining covering more than 5% of the tumour area (>5% IC) [11,12]. This highlights that there are numerous differences between interpretation of these assays: including what cell types are included in the assessment (immune cells or immune cells and tumours cells), the type of inflammatory cells that are included (22C3 allows only lymphocytes and macrophages, whereas SP142 also includes dendritic cells and granulocytes), whether the assessment is of the total number of cells or of the area involved, and finally, the positive cut off values [4,13].

Data from clinical studies found that prior to Atezolizumab, the percentage of tumours with PD-L1 expression of >5% IC varied from 27% (previously untreated tumours in Cisplatin ineligible patients) to either 28% or 33% in those who had prior chemotherapy treatment (28% in metastatic tumour samples, 33% in primary tumour samples) [11,12]. For Pembrolizumab, trials found that 33% (Keynote-052 trial) or 30.3% (Keynote-045 trial) of urothelial carcinoma patients had PD-L1 expressing tumours with a CPS >10 [9,10]. Additionally, a study from Eckstein et al. assessed 251 urothelial carcinomas with four different PD-L1 assays. Their results found that when using the PharmDx assay with the designated cut off of CPS >10, 35.1% of cases were positive. however, when using the SP142 assay with the cut off value of IC >5%, only 16.3% of cases were positive [14].

The existence of multiple different ICB drugs, each with their different compound diagnostic is challenging and inflexible for laboratories in terms of cost, achieving turn around times and preserving patient tissue. This had leads to the drive to assess the interchangeability between different assays. This is a valid consideration that could allow reduce cost, easier testing strategies and preservation of patient tissue [4,13,15].

Several studies have looked at the concordance between multiple different ICB drug assays in urothelial carcinoma. Some compared the concordance between assays using a common criteria, for example, immune cell (IC) value or tumour cell (TC) value rather than each test according to the manufacture guidelines [4,16,17]. Although having a unified assessment method would come with several benefits in terms of ease of assessment and training, and although several studies did find concordance using this method, this is not how the assays were designed and remains unvalidated. As mentioned by Schwamborn et al., the assays are designed to stain for different things; they use different epitopes that are present in different PD-L1 isoforms so a direct comparison is not valid [16]. This is highlighted by the work by Zajac et al., who assessed urothelial carcinoma samples using four available PD-L1 assays. They found that although there was good correlation between SP263, 22C3 and 28-8 assays for both TC and IC PD-L1 staining, when the assays were used with their specific clinical scoring systems, there were substantial differences in scoring, resulting in the conclusion that the appropriately clinically validated algorithm must be used for each drug [4].

A study by Hodgson et al. tested urothelial carcinomas with three commercial kits (SP263, SP142 and 22C3) and assessed each according to the manufacturers algorithm and recommended cut-off value. This study found that the percentages of UC deemed positive in each cancer were 21% using the SP263 clone, 18% using the SP142 clone and 20% using the 22C3 clone. This was deemed a high rate of concordance, however, this was a smaller study of 197 cases of urothelial cancer and it was performed on tissue microarrays, rather than clinical samples [14]. In contrast, as discussed, when Zajac et al. compared the SP263 assay specifically against each of the other three assays (SP142, 22C3 and 28-8), each according to their assay-specific clinically relevant algorithm, their criteria for concordance was not met for any of the assays [4].

In this study we report on the rate of PD-L1 positivity of urothelial carcinomas with the 22C3 and SP142 assay and the clinical correlation between assays. Our aim was to assess whether each assay, if reported according to the relevant manufacturer guidance for urothelial carcinomas, provided reliable information for the alternate drug. For example, in practice, would a 22C3 assay assessment with CPS >10 be reliable to determine if patients would be eligible for Atezolizumab therapy, and would the SP142 assessment with >5% IC be reliable to determine if patients would be eligible for Pembrolizumab therapy?

This data is relevant across cancers with multiple approved ICB drugs because the current inflexibility of the testing strategies is challenging for laboratories. The example provided in this series builds on previous studies comparing concordance between ICB assays in urothelial cancer, when used according to guidelines. Additionally, it sheds light on the tests clinical cross-utility, which may help testing laboratories adjust their strategies to meet oncology requirements more efficiently, while still following the clinically validated manufacturer guidelines.

## Materials and Methods

Our department has been offering PD-L1 testing across various tumour type since 2016, with over 40,000 PD-L1 tests done between 2016 and March 2021. All four compound diagnostics have been validated according to tumour type on the appropriate platforms. Between July 2018 and March 2020, we tested 1,490 advanced urothelial carcinomas for PD-L1 expression.

PD-L1 expression was assessed on formalin-fixed paraffin-embedded (FFPE) tumour samples. Cytology samples, including FFPE clots, were excluded from testing since the preserved architecture of the tissue is required to identify intra-tumoral inflammatory cells to be included in both CPS and IC assessments. In addition, non-invasive carcinomas were not assessed with SP142 assay due to the absence of intra-tumoral inflammatory cells.

Samples were prepared using 4 µm thick sections on Dako slides for the 22C3 assay or on Tomo slides for the Ventana SP142 assay. PD-L1 testing was undertaken using the PD-L1 22C3 PharmDx assay (Agilent Technologies, Santa Clara, CA) on an Autostainer Link 48 and/or the SP142 test (Ventana Medical Systems) on a Benchmark Ultra system. Both assays were validated according the department’s policy for compliance with ISO:15198(2012) standards.

The interpretation of assays was performed by Consultant Histopathologists and one scientist. Reporting members of staff had participated in formal training provided by either Roche Ventana/Dako Agilent, subcontracted specialist training providers or been part of a formal in-house training scheme led by a provider-trained Consultant. The department regularly participates in EQA schemes which assess both the technical staining quality and interpretation of both PD-L1 stains.

22C3 pharmDX assay stained sections were assessed to determine a CPS, with a positive value being CPS >10 as per Pembrolizumab licensing; the maximum CPS was 100. SP142 assay stained sections were assessed to determine an IC score, with a cut-off of 5% of tumour surface positivity as per Atezolizumab licensing.

## Results

There were 1,490 urothelial carcinoma cases submitted to our department for PD-L1 testing between June 2018 and March 2020. Test failure occurred in 89 cases (6%), most of which were due to insufficient material in the sample submitted. Other reasons for failure included submission of cytology samples that are not suitable for PD-L1 assessment (as described previously), samples that lacked an invasive component, or uninterpretable cases due to marked diathermy artifact or necrosis (mostly TURB chippings).

Of the 1,401 samples that had a successful PD-L1 assessment, 621 cases (44%) were tested with both the 22C3 PharmDx and SP142 assays, 492 cases (35%) with 22C3 PharmDx only, and 288 cases (21%) with SP142 only. Therefore, a total of 2022 PD-L1 assays were untaken across these 1,401 samples (1,113 cases were tested with the 22C3 PharmDx assay and 909 cases with the SP142 assay).

Of the cases tested with the 22C3 PharmDx assay, 289/1,113 (26%) cases showed CPS >10 and so were eligible for Pembrolizumab therapy. This figure is a slightly lower rate than shown in the Keynote trials of 30.3% or 33%, and lower than the 35.1% seen by Eckstein et al [9,10,14]. In the cases tested with the SP142 assay, 284/909 (31%) showed IC >5% with the SP142 assay and were eligible for Atezolizumab therapy; this is similar to the range of positivity found in phase 2 trials, and is higher than the 16.3% recorded by Eckstein et al [11,12,14].

**FIGURE 1:**
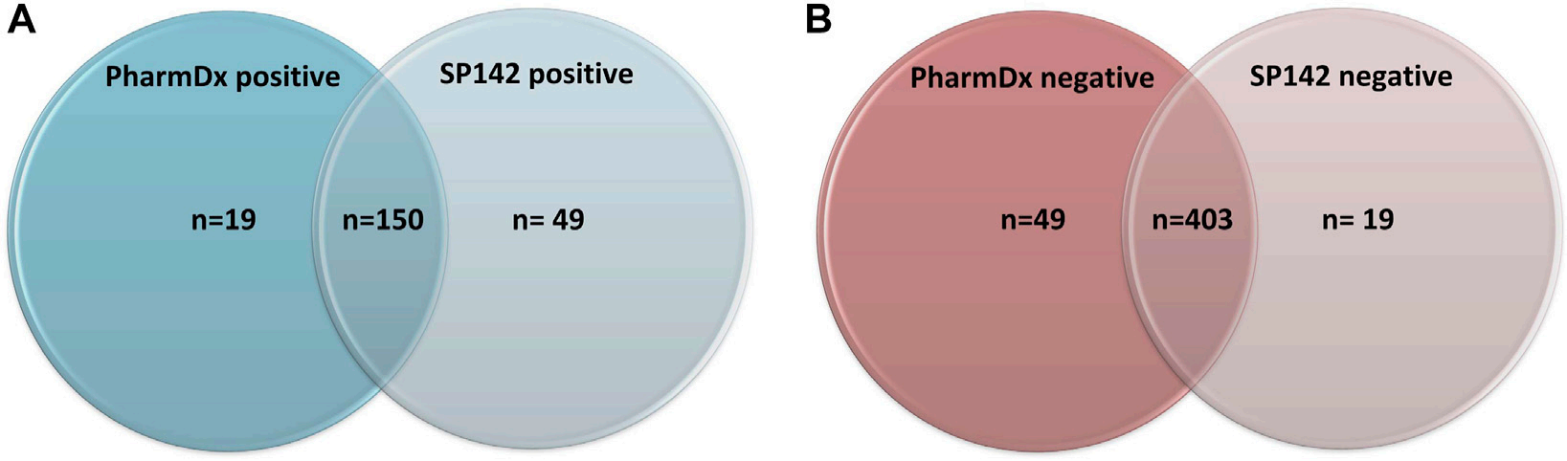
**(A)** Number of positive results across both assays. **(B)** Number of negative results across both assays.

The immunoexpression revealed concordant results (both tests being either positive or negative) in 553/621 cases (89%) and discordant results in 68 cases (11%). Of the concordant results, 403/621 were negative on both assays and 150/553 were positive on both assays (Figure 1A). Of the discordant cases, 49 cases (8%) showed positivity with the SP142 assay (Atezolizumab eligibility) exclusively, and 19 cases (3%) showed positivity with the 22C3 PharmDx assay (Pembrolizumab eligibility) exclusively.

## Discussion

Worldwide, PD-L1 testing workflows for urothelial carcinomas in routine practice are complex; they potentially require two platforms, multiple assays and appropriately trained pathologists and scientists. Our data provides some insight on how to rationalise PD-L1 testing before ICB therapy in order to improve efficiency.

We have obtained data on the clinical discrepancy between both PD-L1 assays, rather than merely comparing the pattern of PD-L1 expression between the assays. This point is vital because the assays have been designed in specific conditions and have different staining characteristics. Indeed, in practice, we see significant differences in the intensity and the proportion of both tumour and inflammatory cell staining depending on which assay has been used. Therefore, it would not be appropriate to apply a cut-off of CPS >10 on sections stained with SP142 assay or a cut-off of >5% IC on sections stained with the 22C3 PharmDx assay. Instead, it is essential to adhere to the guidance provided by the companion diagnostics to ensure accurate results and to maintain the comparability of results among different laboratories.

Our data shows that PD-L1 testing is feasible in routine practice with a low rate of failure. Our positivity rate for each assay is very similar to that in Keynote trials for 22C3PharmDx and pre-licensing studies for SP142 assay. Furthermore, the positivity seen with the SP142 assay in our study was significantly higher than seen by Eckstein et al and so does not support their conclusion that using the SP142 assay would detect fewer patients eligible for PD-L1 therapy [14]. Additionally, PD-L1 testing with both assays on 621 tumours found that the clinical discrepancy was 11%. Unfortunately, no statistical analysis could be performed because of the low number of discrepant cases; however, a discrepancy of 11% is too high to be ignored and limits the clinical cross-utility between tests.

Interestingly, when two ICB drugs were licensed for use in urothelial cancer, dual 22C3 SP142 PD-L1 expression testing was only requested in 44% of cases. This value is lower than would be expected and may be because as PD-L1 testing was the first molecular test to be mandatory before targeted therapy for urothelial carcinomas, so the teams of clinicians and pathologists may not have been familiar with the pathway, workflow and logistics for requesting and collecting results of molecular tests. This is important to address to avoid introducing bias into the test selection.

Moving forward, since we have demonstrated limited cross-utility between assays, laboratories must find other approaches in scenarios where multiple ICB drugs are available. One approach laboratories could employ to avoid testing all tumours with multiple assays is to implement sequential testing. This approach would require a case-by-case assessment, with the first assay used guided by the oncologists’ preferences between the available drugs. This could represent an acceptable and sensible option despite requiring complex logistics.

To facilitate logistics in our lab, when multiple ICB drugs were approved for urothelial cancer we used a detailed request form, which described both PD-L1 testing assays and which drug corresponds to each assay. This clarity is essential as the choice of drug and, by extension, the selection of the PD-L1 assay is driven by oncologists, not the testing laboratory (as this choice is based on clinical criteria including modalities of prescription and expected potential side effects).

In conclusion, it is paramount to utilise PD-L1 assays according to the manufacturer guidelines to allow result accuracy and standardisation. Furthermore, due to limited cross utility the appropriate assay must be used for each different ICB drug. The factors contributing to the discrepancy between assays remain undefined and further research into this is warranted, particularly to meet the continued drive to improve the testing strategies for ICB drugs. Finally, further research into whether discordant assay results lead to any clinical differences in patient response to different ICB would be valuable to the field. Together, this would increase our understanding of how best to test and treat patients with different ICB drugs, ensuring maximum patient benefit is achieved.

## Data Availability

The raw data supporting the conclusion of this article will be made available by the authors, without undue reservation.

## References

[1] Cheung CC Barnes P Bigras G Boerner S Butany J Calabrese F Fit-for-purpose PD-L1 Biomarker Testing for Patient Selection in Immuno-Oncology: Guidelines for Clinical Laboratories from the Canadian Association of Pathologists-Association Canadienne Des Pathologistes (CAP-ACP). Appl Immunohistochem Mol Morphol (2019) 27(10):699–714. 10.1097/pai.0000000000000800 31584451 PMC6887625

[2] Eckstein M Cimadamore A Hartmann A Lopez-Beltran A Cheng L Scarpelli M PD-L1 Assessment in Urothelial Carcinoma: a Practical Approach. Ann Transl Med (2019) 7:690–22. 10.21037/atm.2019.10.24 31930091 PMC6944605

[3] Powles T Walker J Andrew Williams J Bellmunt J. The Evolving Role of PD-L1 Testing in Patients with Metastatic Urothelial Carcinoma. Cancer Treat Rev (2020) 82:101925. 10.1016/j.ctrv.2019.101925 31785413

[4] Zajac M Scott M Ratcliffe M Scorer P Barker C Al-Masri H Concordance Among Four Commercially Available, Validated Programmed Cell Death Ligand-1 Assays in Urothelial Carcinoma. Diagn Pathol (2019) 14(1):99–10. 10.1186/s13000-019-0873-6 31477145 PMC6720992

[5] National Institute for Health and Care Excellence. Atezolizumab for Untreated PD-L1-Positive Advanced Urothelial Cancer when Cisplatin Is Unsuitable. last update (2021). Available at https://www.nice.org.uk/guidance/ta739/chapter/1-Recommendations Oct 27 (Accessed February 28, 2022) 40802807

[6] National Institute for Health and Care Excellence. Atezolizumab for Treating Locally Advanced or Metastatic Urothelial Carcinoma after Platinum-Containing Chemotherapy. last update (2018). Available at https://www.nice.org.uk/guidance/ta525/chapter/1-Recommendations June 13 (Accessed February 28, 2022) 41397083

[7] European Medicines Agency, -last Update, Tecentriq/Atezolizumab. Summary of Product Characteristics. last update (2021). Available at: https://www.ema.europa.eu/en/documents/product-information/tecentriq-epar-product-information_en.pdf Nov 10 (Accessed February 28, 2022)

[8] European Medicines Agency. Keytruda/Pembrolizumab. Summary of Product Characteristics. last update (2022). Available at https://www.ema.europa.eu/en/documents/product-information/keytruda-epar-product-information_en.pdf Jan 11 (Accessed February 28, 2022)

[9] Balar AV Castellano D O'Donnell PH Grivas P Vuky J Powles T First-line Pembrolizumab in Cisplatin-Ineligible Patients with Locally Advanced and Unresectable or Metastatic Urothelial Cancer (KEYNOTE-052): a Multicentre, Single-Arm, Phase 2 Study. Lancet Oncol (2017) 18(11):1483–92. 10.1016/s1470-2045(17)30616-2 28967485

[10] Fradet Y Bellmunt J Vaughn DJ Lee JL Fong L Vogelzang NJ Randomized Phase III KEYNOTE-045 Trial of Pembrolizumab versus Paclitaxel, Docetaxel, or Vinflunine in Recurrent Advanced Urothelial Cancer: Results of >2 Years of Follow-Up. Ann Oncol (2019) 30(6):970–6. 10.1093/annonc/mdz127 31050707 PMC6594457

[11] Balar AV Galsky MD Rosenberg JE Powles T Petrylak DP Bellmunt J Atezolizumab as First-Line Treatment in Cisplatin-Ineligible Patients with Locally Advanced and Metastatic Urothelial Carcinoma: a Single-Arm, Multicentre, Phase 2 Trial. The Lancet (2017) 389(10064):67–76. 10.1016/s0140-6736(16)32455-2 PMC5568632 27939400

[12] Rosenberg JE Hoffman-Censits J Powles T Van Der Heijden MS Balar AV Necchi A Atezolizumab in Patients with Locally Advanced and Metastatic Urothelial Carcinoma Who Have Progressed Following Treatment with Platinum-Based Chemotherapy: a Single-Arm, Multicentre, Phase 2 Trial. The Lancet (2016) 387(10031):1909–20. 10.1016/s0140-6736(16)00561-4 PMC5480242 26952546

[13] Lee KS Choe G. Programmed Cell Death-Ligand 1 Assessment in Urothelial Carcinoma: Prospect and Limitation. J Pathol Transl Med (2021) 55(3):163–70. 10.4132/jptm.2021.02.22 33823566 PMC8141973

[14] Eckstein M Erben P Kriegmair MC Worst TS Weiss C-A Wirtz RM Performance of the Food and Drug Administration/EMA-Approved Programmed Cell Death Ligand-1 Assays in Urothelial Carcinoma with Emphasis on Therapy Stratification for First-Line Use of Atezolizumab and Pembrolizumab. Eur J Cancer (2019) 106:234–43. 10.1016/j.ejca.2018.11.007 30528808

[15] Hodgson A Slodkowska E Jungbluth A Liu SK Vesprini D Enepekides D PD-L1 Immunohistochemistry Assay Concordance in Urothelial Carcinoma of the Bladder and Hypopharyngeal Squamous Cell Carcinoma. Am J Surg Pathol (2018) 42(8):1059–66. 10.1097/pas.0000000000001084 29750666 PMC6750743

[16] Schwamborn K Ammann JU Knüchel R Hartmann A Baretton G Lasitschka F Multicentric Analytical Comparability Study of Programmed Death-Ligand 1 Expression on Tumor-Infiltrating Immune Cells and Tumor Cells in Urothelial Bladder Cancer Using Four Clinically Developed Immunohistochemistry Assays. Virchows Arch (2019) 475(5):599–608. 10.1007/s00428-019-02610-z 31267201 PMC6861354

[17] Tretiakova M Fulton R Kocherginsky M Long T Ussakli C Antic T Concordance Study of PD-L1 Expression in Primary and Metastatic Bladder Carcinomas: Comparison of Four Commonly Used Antibodies and RNA Expression. Mod Pathol (2018) 31(4):623–32. 10.1038/modpathol.2017.188 29271413

